# Concerning Editors

**Published:** 1856-02

**Authors:** 


### CONCERNING EDITORS.

*The Evangelical Magazine* has an article suggesting the propriety of "Prayer on behalf of the Editors of Christian Journals." Mere human reason is an enemy of religion; but true philanthropy goes hand in hand with the Christianity of the Bible. As philosophers, let us look at this subject with the seriousness which becomes it. If there is a single doctrine in the Bible reiterated in multifold forms, it is this, *That the blessings of the Almighty must be looked for authoritatively in the performance of duty;* that is to say, in the rational employment of those means which are calculated to accomplish the end. We need not pray to be fed, unless we use means to procure food. The Church must first get a suitable man to be an editor; to be such, he must be pious, educated, healthy; neither of these three requisites has just precedence of the others. A man may be an educated infidel; he may be a pious fool; he may have piety and education, and yet dyspepsia may make him a ranting fanatic, a raving madman or a dogmatic driveller. Many an editor needs a pill more than a prayer. A worn out preacher is not fit for an editor; no sick man is. There are editors of religious newspapers, whose piety we dare not question—on the contrary, we feel this moment, as if we would gladly exchange it for our own—and whose mental culture far exceeds ours, but whose intolerance of opinion, whose forwardness to publish the fallings and the failings of those of a different sect, whose impatient uncourteousness in editorial controversies, whose free dealings in ungentlemanly personalities, we would not possess for all creation. In fact, a most observable difference between the secular and the religious press, is this—that too many of the latter seem to riot in the freedom they feel, of security against being *called out*, to answer for the application of epithets, which men of the world would meet with a bullet.

It is a notorious fact, that the principal editor of one of the largest and most extensively circulated *religious* weeklies, is personally, in private life, one of the most amiable of men; but as an editor, hurls against his antagonist, whether a public man or of private station, epithets so hard, so severe, so unfeeling, so unforbearing, so vindictive, that we all see he needs only the power, to make him an inquisitor. Taking this man's piety for granted, in connection with his known previous amiability of temper, the only solution of the incongruity is, that severe application has made him dyspeptic; and this it is, that has vinegarized the whole man, which has made him a lost pleiad among the delightful characters of his time. Many men, who are so fortunate as to have no taste for liquor, have no difficulty in declaring, that any man who takes daily a glass of wine or brandy can have no religion; and yet these same men will over-eat themselves three times a day, until the stomach, constitution, temper, health, all are ruined, and the remainder of their days are spent in scribbling sickly sentimentalities for other people. To make, then, the religious press of this country what it ought to be—the handmaid of the Pastor and the Missionary—supply each paper with an editor who has the most vigorous health; who has the learning of an Anthon, the piety of a Payson, and the bonhommie of a Sydney Smith—with a salary, prompt and unconditional, of five thousand a year. Such a man is well worth it, and one of the best economies of the Church would be, to supply such an one to every religious newspaper in the country.

On "*second thoughts*," WE have good health, and have Sydney's benevolent good nature, besides being nearer orthodox than he. As to learning, we used to have some skill in Hebrew, Greek, Latin, Spanish, Italian, and German, besides mother English; and our readers have seen with what facility we can take a phrase out of a dictionary of quotations, and write up to it. But we confess to some rustiness in all these tongues—to have arrived at an age when we feel most at home in the language of——dollars. Any good salaried editorial vacancy? Only two conditions—Salary sure!—No gag.

